# Evaluating change in a pressured healthcare system: a cross-sectional study of implementation outcomes using routine data indicators and proxies

**DOI:** 10.1186/s43058-023-00471-x

**Published:** 2023-08-16

**Authors:** Andria Hanbury, Nyasha Mafirakureva, Nicola Chicken, Liam Bailey

**Affiliations:** 1 Yorkshire and Humber Applied Research Collaboration, Bradford Institute for Health Research, Bradford, UK; 2 School of Health and Related Research, University of Sheffield, Sheffield, UK; 3 Yorkshire and Humber Academic Health Science Network, Wakefield, UK; 4 Yorkshire Quality and Safety Research Group, Bradford Institute for Health Research, Bradford, UK

**Keywords:** Implementation evaluation, Routine data, Downloaded data, Administrative data, Indicators, Implementation outcomes framework, Cost, Adoption, Feasibility, Sustainability, Fidelity

## Abstract

**Background:**

Implementation evaluation should focus on implementation success, guided by theories and frameworks. With high staff vacancies in the health services, it is important to consider pragmatic methods of data collection for implementation evaluation. This paper presents a cross-sectional rapid evaluation of a handheld medical device designed for remote examinations, piloted in Northern England. By using downloaded device data and administrative records mapped to domains from the implementation outcomes framework, this evaluation offers a pragmatic example of assessing implementation success.

**Methods:**

The pilot design was pragmatic: sites volunteered, decided which services to use the device in, and launched when ready. The pilot and evaluation together lasted 1 year. Data was downloaded from the devices, and administrative records for the pilot accessed. Variables were mapped to five of the implementation outcomes, after reviewing with the device manufacturer and pilot team to assess robustness.

**Results:**

*N*=352 care episodes were recorded using the device with 223 patients. Out of 19 sites ‘signed up’ to the pilot, 5 launched and delivered 10 of 35 proposed projects: a site and project adoption rate of 26 and 29%, respectively. Six sites signed up to an extension period; three had launched and three had not during the original timelines, indicating some sustainability. Feasibility was high, with only one in seven care episodes needing to be repeated due to poor device quality or error (sound/audio/internet). Fidelity of device usage was low for two of the eight available device examinations. Device and staffing costs were high but potential cost savings were attributable to fewer in-person appointments.

**Conclusions:**

Through using device and administrative data, this evaluation minimised burden on busy healthcare staff yet was still guided by an evaluation framework. Five out of the eight implementation outcomes were measured, including sustainability and costs. The findings give insight into implementation challenges, particularly around adoption. For future research, it is recommended to engage with staff to prioritise outcome measurements and to focus on meaningful interpretation of indicators.

**Supplementary Information:**

The online version contains supplementary material available at 10.1186/s43058-023-00471-x.

Contributions to the literature

- Implementation research is currently taking place within a health care system with high staff vacancies.
- This paper places a timely emphasis on the ease of using downloaded digital device and administrative data within the context of a remote monitoring evaluation, which can still be used with a theory or framework-led evaluation.
- Limitations of such data for implementation evaluation are discussed, including meaningful interpretation, which are suggested for future research consideration.

## Background

Implementation success for healthcare interventions is crucial for the long-term sustainability of interventions, but it is often overlooked in favour of evaluating intervention effectiveness. Selecting the appropriate outcome measures is essential for evaluating implementation success as it allows for a comprehensive understanding of the implementation process and aids in the meaningful comparison of results across different studies and settings. Assessing implementation outcomes is vital for determining the overall success of an intervention as it provides a holistic understanding of the intervention’s performance and ability to be effectively implemented, adopted, and sustained in real-world settings [21]. Even if an intervention is highly effective, poor implementation can impede its overall effectiveness by limiting the number of people who are exposed to it or by delivering a suboptimal version of it due to low fidelity.

Over the past two decades, there has been a growing recognition of the importance of using theories and frameworks to guide health care intervention development, implementation, and evaluation. This facilitates a more targeted/systematic approach and helps to develop an evidence base. There are numerous papers on implementation determinants (barriers and facilitators) (e.g. [7]) and on the theories (e.g. diffusion of innovation, [22], models (e.g. COM-B, [14]), and frameworks (e.g. consolidated framework for implementation research, [4]) to guide exploration of these. And, they can also be used to guide evaluation design; for example, measuring barriers and facilitators pre and post intervention delivery to assess how well they have been targeted. Nilsen [17] provides a helpful overview and classification of the models, theories, and frameworks, for example, distinguishing between ‘determinant frameworks’ which provide a summary of implementation barriers and facilitators, ‘classic’ and ‘implementation’ (specific) theories which propose relationships between them and outcomes, and *evaluation frameworks.* Evaluation frameworks, such as Proctor et al.’s [21] implementation outcomes framework, focus instead on outcomes related to implementation success. Proctor et al. [21] propose the following eight implementation outcomes to be of importance. *Adoption* is defined as the uptake of a new intervention or programme. *Penetration* is the integration of the intervention within routine practice. *Feasibility* refers to the extent to which an innovation can be carried out in the given setting. *Fidelity* is defined as the degree to which an intervention is implemented as intended. *Perceived acceptability* refers to the level of utility the intervention has among the target population or stakeholders. *Perceived appropriateness* is the level of relevance, suitability, or fit of an intervention in the context of the organisation or target population. *Sustainability* is defined as the ability of an intervention to continue and maintain its effects over time. Finally, *Cost* refers to the financial resources required to implement and maintain an intervention, including innovation cost, the cost of the implementation strategy, and the location of service delivery.

Robust implementation evaluation can be challenging when working with healthcare organisations that have high staff vacancies (10.8% vacancy rate for registered nurses between April and December 2022, up from 10.2% the previous year, [15]). This can have knock-on effects for staff workload, wellbeing, and retention; issues intensified during the Covid-19 pandemic and addressed through the NHS People Plan [16] which focuses on recruitment and training, new ways of working, and developing an inclusive organisational culture. Implementation evaluators need to be cognisant of the challenges faced by healthcare organisations and take them into consideration. This involves striking a balance between engaging with health care staff to optimise implementation efforts, including evaluation, while trying to keep the additional workload manageable. The need to reduce burden for implementers and researchers has led to a growing focus on pragmatic measurement (e.g. [20, 25].

One pragmatic approach to implementation evaluation is making use of routine indicators, administrative data and, with the increasing attention on digital innovations in health care, downloadable data from health care devices. The latter arguably overcomes some of the shortcomings of traditional routine data such as electronic health records—including missing and unreliable data due to error in data entry [19]—through being device-specific and automated—e.g. a remote monitoring device providing data on device usage and duration of usage. We suggest that quantitative indicators such as these should be the first ‘port of call’ when designing an evaluation. This approach does not exclude the use of surveys and qualitative methods, as they provide valuable insights from the perspective of healthcare professionals and patients. Instead the emphasis is on first checking what indicators can be collected using routine or admin or device-downloaded data, and exploring for proxy measures where direct measures are not available, before then considering other ways of collecting data. Indeed, adopting a mixed approach that combines the use of indicators with short, targeted interviews allows for the exploration of constructs that may not be easily captured by indicators and routine data, while also keeping the length of interviews to a minimum. Making use of indicators not only reduces the demands on front line health care staff but also enables easier longer-term assessment of intervention sustainability through routine monitoring and potential benchmarking [11]. If existing indicators or device-downloadable data are available, the burden on operational health care staff (such as computing and quality improvement) should also be kept low.

Routine data usage in evaluation is not new, but has less frequently been theory/framework based, and where it has, has focussed on some implementation outcomes more than others. For example, a scoping review of quantitative indicators used in implementation research and evaluation [29] identified only 10 papers published between 2008 and 2018 that used such indicators to monitor intervention quality or implementation success. Of these, 5 papers used a theory or framework to guide indicator selection. When the 67 indicators across the 10 papers were mapped to Proctor et al.’s implementation outcomes framework to assess coverage of the different outcomes, fidelity and penetration rates were found to be most frequently reported, whereas appropriateness and sustainability were not measured. Costs, feasibility, adoption, and acceptability were also less frequently measured [29].

In this paper, we present an example of the administrative and downloadable device data used to evaluate implementation of a handheld remote monitoring device—‘TytoCare’—which is able to perform clinical grade audio and visual examinations of the heart, lungs, ear, throat, and skin. The examinations can be carried out by patients/carers themselves, following instructions generated by the device using the ‘Home’ version of the device, or carried out by health care professionals using the ‘Pro’ version of the device. The device was piloted across the Yorkshire and Humber region, England, for a programme of work on remote monitoring and virtual wards led by the Yorkshire and Humber Academic Health Science Network (Yorkshire and Humber AHSN). Remote monitoring is a crucial area of development in healthcare, enabling patients’ health to be monitored in their own homes. Remote monitoring is associated with better patient experiences [5, 18] and quality of care [2, 6, 27], more efficient use of healthcare professionals’ time [3], and wider system and societal benefits [1, 23, 24].

The data collected in this evaluation were used in conjunction with a small sample of qualitative interviews, analysed using the framework-guided rapid analysis approach [8]. The focus of this paper is on the device-downloaded and administrative data, with the aim of highlighting the issues encountered and areas for future research. Each implementation outcome measured was mapped to Proctor et al.’s [21] implementation outcomes framework.

## Methods

The pilot launch began in June 2021 and ran until June 2022, spanning the second wave of the Covid-19 pandemic. To allow for additional data collection, a 3-month extension period was added to the pilot, ending in September 2022. However, data from this extension period is not included in this paper, as it falls outside of the scope and timelines of the original evaluation.

### The device

**Fig. 1:**
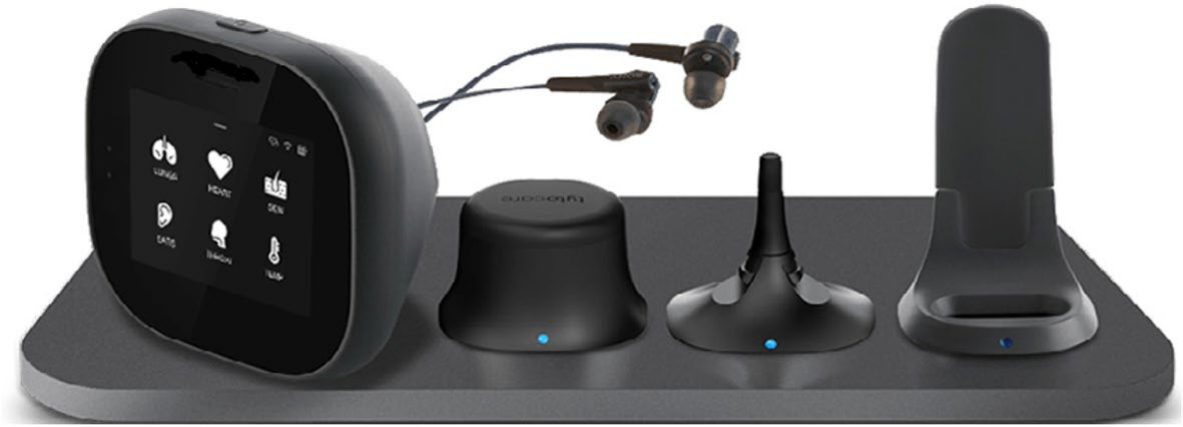
The TytoCare device

The TytoCare device (Fig. 1) is a handheld remote monitoring device, able to perform clinical grade audio and visual examinations of the heart, lungs, ear, throat, and skin. The device has a camera for skin examinations and examination of the tonsils (a tongue depressor attachment aids this), temperature sensor, a stethoscope to examine lung and heart functioning, and an otoscope to capture images of the ear drum. As such, the device has potential to be used in a range of different care settings for monitoring of a range of different conditions. The ‘Home’ version of the device sits in the patient’s home and enables these examinations to be performed by the patient during a ‘live’ consultation (via a platform, similar to other online video systems), or alternatively, performed and submitted ‘offline/outside of a consultation’ by the patient for later review by the healthcare team. The device provides user-friendly instructions for patients for how to perform each examination as appropriate. The ‘Pro’ version of the device is designed for healthcare professionals, enabling cross site collaborations (for example, between general and specialised services) and their performing examinations for patients within the community, for example, a community nurse using the device in a care home and enabling a real-time or ‘offline’ review by a hospital specialist. The device requires internet connection for the live consultations and for the submission of examinations data.

### Recruitment of sites

Sites (health care organisations, such as NHS foundation trusts and care homes) were based within three integrated care systems within the Yorkshire and Humber region of Northern England. No other restrictions were placed on participation. The Yorkshire and Humber AHSN (one of fifteen organisations established by NHS England in 2013 to spread health and social care innovation) released a call for interested healthcare sites to participate. Sites were free to choose the care setting and conditions to pilot in and with, and the most appropriate ‘device usage’ for them including which device to use. Device usage could include virtual ward set-up, e.g. monitoring patients at home or within a care home or cross site collaborations between general and specialist teams. A single site could host more than one pilot project; each care setting and chosen device usage was set up as a separate pilot project.

### Context and data sources

Device costs were centrally paid for and centralised support was provided for implementation. The cost to sites was, therefore, restricted to staffing costs (using existing staff time) for planning the pilot at their site (device usage, care setting, benefit realisation), project management, and attending training. The evaluation team were not involved in implementation, which was completed by project teams and the commissioners/payers, the Yorkshire and Humber AHSN and the device manufacturer. The process comprised:

- Scoping—project team, identifying aims, anticipated outcomes and benefits, scope, pathway/workflow design
- Governance completion—Clinical Safety, Equality Impact Assessment, Data Protection Impact Assessment, Data Protection Contract
- Network and IT testing and set-up
- Training—clinicians, other health care professionals, administrative teams, patients, families, carers, first-line support
- End to end testing
- Communications to all required stakeholders
- Final sign off and ‘Go-Live’
- Post-live support

Available data for evaluation was:

- Data downloaded from the device at pilot sites, detailing each episode of care where the device was used. Variables were as follows: whether the entry was a demonstration/test run, project, site, patient and clinician identifiers, date of care episode, duration, type of device (Home or Pro), the type of examination performed (ear, heart, lung, temperature, skin) and whether it was done using the device (for example, some examinations like temperature check could be done using standard equipment or the device). Device-downloaded data also included clinician responses to ‘pop-up’ questions that were programmed to appear after a real-time consultation or after their reviewing ‘offline’ submitted examination data. These asked clinicians to:
  1. Indicate whether they considered the contact to have avoided a face to face appointment/accident and emergency visit as appropriate (yes/no)
  2. Rate overall satisfaction and the audio and visual quality (on a 5-point scale)
  3. Indicate whether the appointment had to be repeated due to poor quality (e.g. sound/visual, internet) or error (yes/no).

Clinicians were able to skip the questions.

- Administrative data held by the pilot team:
  - ◦ Records of interested sites, sites that went on to launch, sites that signed up for an extension period, associated dates.
  - ◦ Documents detailing device licencing (numbers and cost), device type (Home or Pro) and how the device was being used (for direct consultations or offline reviews of examinations).
- Training records: staff and patients (the latter, only applicable to patients coming into contact with the home version of the device).

The data was downloaded once at pilot end and supplied to the evaluation team by the device manufacturer. The data can be downloaded directly from a dashboard by clinicians/trusts/providers, for in-house monitoring.

Table 1 summarises the data sources and specific variables/indicators, and Table 2 summarises how these were combined (as appropriate) to provide implementation outcome measures mapped to Proctor et al.’s framework. Adoption was measured through comparing the number of sites and projects expressing an interest in the pilot (administrative records) with the number that went on to launch (downloaded data). Penetration was the number of clinicians trained (administrative data) who used the device (downloaded data). Feasibility was via a proxy: the proportion of care episodes which had to be repeated due to poor quality (e.g. sound/visual/internet) or error (using the third clinician’s pop-up question). Fidelity was also via a proxy: the proportion of examinations performed using the device (as intended for the pilot) versus not (e.g. checking heart rate with a standalone stethoscope rather than the device). Sustainability was estimated via the length of time projects and sites used the device and whether they signed up to the extension period.

**Table 1:** Data sources, variables and indicators details

**Data source**	**Variable**	**Description**
**Device collected data: details for every recorded episode of care using the device.**	Project ID	Each project was named in the dataset.
	Site ID	Each site (NHS Trust) was named in the dataset. Some sites hosted multiple projects, hence the need for a project identifier as well as a site identifier.
	Device type	Home (kept in patient’s home, controlled by the patient) or Pro device (held and controlled by the health care professional).
	Type of care episode/device usage	Direct online consultation; off line examination by patient (Home device) sent for offline review; off line examination by health care professional (Pro device) sent for offline review. This was used for the costing analysis only.
	Demonstration	Whether the entry was a demonstration during testing/set-up or a genuine care episode. Demonstrations were removed during data cleaning.
	Duration of contact	Length of care episode, in minutes. This was used for the costing analysis only.
	Type of clinician	Health care assistant; GP practice nurse; band 7 hospital nurse; GP; speciality registrar; Consultant (medic). This was used for the costing analysis only.
	Pseudonymised clinician identifier	Clinicians could appear more than once in the dataset; this variable enabled us to count the number of different clinicians using the devices without identifying who they were.
	Examination (heart, or heart rate, lung, skin, throat, ear, temperature)	Type of examination performed (but not necessarily using the device as clinicians could perform an examination using their own equipment and then enter this into the record).
	Examination ordering	Patients could have multiple examinations per care episode and so separate variables were created for 1st, 2nd, 3rd, *n* examinations to enable us to capture each examination separately, which was easier for analysis.
	Whether examination performed using device or not.	For each examination above, we created a separate variable identifying whether it was done using the device or not, which was indicated in the dataset via a variable titled ‘counter’, dummy coded as ‘1’ for yes (using device) and ‘2’ for no (‘not using device’). E.g. it was possible for clinicians to use their own equipment and enter the data in to the patients’ record.
	Real-time pop-up questions built into the device. These covered: (1) Ratings of audio and visual quality (2) Assessment as to whether the contact avoided a face to face appointment (yes/no) (3) Whether the examination had to be repeated due to poor quality.	#1 was via a 5-point Likert scale, #2 and #3 via a ‘yes/no’ response.
Qualitative interviews (not covered in this paper)		Perceptions of acceptability and appropriateness.
		Perceptions of sustainability.
		Perceptions of equality/inequalities impact.
**Administrative data.**	Project details	Care setting; specified device usage (e.g. ‘to remotely monitor paediatric patients following discharge’).
	Number of health care professionals trained	Collected at project level.
	Number of patients trained to use the Home device (where applicable)	For patients, training was only necessary for those needing to use the Home device.
	Sites and projects progress: • Signed up but not live (failed to launch). • Live. • Signed up to pilot extension.	In the analysis, signed up but not live were considered sites that had expressed an interest but had failed to launch, given that the download happened after the pilot end.
	Device licencing (numbers and cost via procurement prices) and device uses.

**Table 2:** Mapping of variables and indicators to implementation outcomes and how calculated

Implementation outcome	Variables used	How calculated
Adoption	Project ID (device data) and project progress (admin data) Site ID (device data) and site progress (admin data)	Number of projects that expressed interest in the pilot who actually ‘went live’ Number of sites that expressed interest in the pilot who had at least one project that ‘went live’
Penetration	Pseudonymised clinician identifier (device data) and number of trained clinicians (admin data)	Number of trained clinicians divided by number of clinicians using the device
	Pseudonymised patient identifier (device data) and number of trained patients (admin data, Home device only)	Number of trained patients divided by number of patients using the device (Home device only)
Sustainability	Site and project ID (device data) and whether signed up to pilot extension (admin data)	Frequency count of the number of sites and projects that signed up to the pilot extension period.
Fidelity	Examination and whether performed using device (device data)	Frequency count and proportion of examinations performed using the device (as planned) versus not (using different equipment).
Feasibility	Real-time clinician pop-up question #3: whether the examination had to be repeated due to poor quality	Frequency count of number and proportion of care episodes needing to be repeated due to poor quality.
Cost	Site and project ID, duration of contact, type of contact, clinician type, device type (all device data) and site and project progress, and clinicians and patients trained and device licencing (all admin data).	Please see Additional file 1 for costing details.

The process for selecting these variables comprised the following 3 stages:

1. Reviewing the variables available in the device-downloadable data and the administrative data.
2. Selecting those that could be mapped to implementation outcomes.
3. Discussion with the device manufacturing company and the pilot team regarding the robustness of the indicators: did they measure what they appeared or purported to measure (validity), and reliably? Any issues that may reduce the reliability or validity of the measures were identified and followed by discussions to either address the issue if feasible, or discount the variable if too significant.
  There was one variable dropped from the evaluation based on stage 3, detailed under the subheading ‘variable selection’ in the results.

### Data cleaning and analysis

Data was exported into a statistical software programme, RStudio, for cleaning and analysis. Missing data was treated as missing data, and care episodes identified as demonstration/test runs linked to device launch or training were excluded from the analysis.

Analysis comprised:

1. Describing the sample (number of care episodes, number of sites and projects represented)
2. Running a frequency count for the one implementation outcome where no combining of variables was required: feasibility.
3. Combining variables where needed to generate the outcome measure (see Table 2: e.g. calculating adoption, sustainability, fidelity) and running frequency counts on the newly created variable/outcome.
4. The costs analysis. Costs were estimated from the perspective of the NHS and personal social services. Resources used in the pilot were identified by mapping the steps undertaken to plan and implement the pilot. This was done through project document review, speaking to project staff and a review of care pathways. An additional costing method file provides more detail regarding the methodology and calculation of device costs and project cost savings (see Additional file 1).

## Results

### Variable selection

Using the three-stage process to select variables for analysis, one planned outcome measure—penetration—was removed. This was due to concerns regarding the accuracy of the administrative data which recorded the number of patients and clinicians trained. Some sites had not kept their training data up to date, making the numbers unreliable (for example, more patients using the device than had been trained to use them which was not possible due to training being a prerequisite for receiving a home device).

### The sample: sites, projects, and number of care episodes

A total of *N*=352 care episodes were conducted using the device, with 223 patients and 26 clinicians participating. Of the 19 sites that had signed up to participate in the pilot, 5 launched the device and hosted a total of 10 pilot projects (out of the original 35 proposed). Of these 10, 1 site used the device with working age adults in an emergency care department, 4 with older people (of which 1 was a GP practice and 3 were care homes), and 5—all acute care trusts—with paediatric patients and their families. The device was used to monitor respiratory patients for 3 projects (all paediatric settings), a range of conditions for 5 projects (the older people care settings and the emergency department setting), cleft lip and palate patients for 1 (paediatric) and palliative care for 1 (paediatric). Details regarding the sites, projects, device type and the way they used the device are summarised in Table 3. Codes are provided in all of the tables to avoid identifying sites and their projects, based on agreements for the evaluation.

**Table 3:** The 10 pilot projects launched from the five sites, their care setting, device usage, conditions/pathway used for, what was being monitored and frequency of monitoring/consultations

**Site (project)**	**Care setting**	**Device usage**	**Conditions/pathway recommended for**	**What was being monitored**	**Frequency of monitoring/consultations**
**Site A (Project 1)**	GP Practice, older adults	Remote GP appointments for older people in their own homes. Pro device	Various conditions Used to take clinical observations and for remote GP appointments when a home visit request is received into the central Home Visiting Hub	Various observations as needed	Ad hoc when GP home visit requested by patient/carer
**Site A (project 2)**	Care Home, older adults	Remote GP appointments for older people in care homes. Pro device	Various conditions Used to take clinical observations and for remote GP appointments when usually a telephone appointment or in-person care home visit would take place	Various observations as needed	Ad hoc when GP telephone appointment or home visit requested by care home
**Site A (project 3)**	Care Home, older adults	Remote GP appointments for older people in care homes. Pro device	Various conditions Used to take clinical observations and for remote GP appointments when usually a telephone appointment or in-person care home visit would take place	Various observations as needed	Ad hoc when GP telephone appointment or home visit requested by care home
**Site A (Project 4)**	Care Home. Older adults	Remote GP appointments for older people in care homes. Pro device	Various conditions Used to take clinical observations and for remote GP appointments when usually a telephone appointment or in-person care home visit would take place	Various observations as needed	Ad hoc when GP telephone appointment or home visit requested by care home
**Site B (Project 5)**	Acute Hospital Trust: tertiary care, paediatrics	Remote hospital appointments for children in their own homes. Home device	Cleft Lip and Palate	Camera images and videos of patient’s mouth	As required for diagnosis and treatment/surgery pathway
**Site C (Project 6)**	Acute Hospital Trust, paediatrics	Remote nurse visits for children in their own homes. Devices with patients & families for virtual clinics in their own homes. Home and Pro devices	Respiratory conditions that require home ventilation	Chest Ears Throat Abdomen Temperature	Routine (3/6/12 month depending on patient cohort) Or ad hoc as required
**Site C (Project 7)**	Acute Hospital Trust, paediatrics	Remote healthcare professional visits for children in their own homes Home and Pro devices	Palliative care	Video impression Chest sounds Other exams as required	Routine (Fortnightly, monthly or less frequently depending on patient needs) Or ad hoc as required
**Site D (project 8)**	Acute trust: Emergency Department, working age adults	Emergency Department consultant providing consultations remotely. Pro device	Various conditions Used to enable ED Consultant to provide consultations remotely (Patient in ED with Health Care Assistant, Consultant remote)	Various observations as needed	72 ED consultations conducted
**Site D (Project 9)**	Acute Trust, paediatrics	Devices with patients & families for virtual clinics in their own home. Home device.	Respiratory conditions	Chest Ears Throat Temperature	Ad hoc when patient/family contact consultant for review
**Site E (project 10)**	Acute Trust, paediatrics	Devices with patients & families for monitoring their condition in their own homes. Home device	Respiratory conditions	Chest Ears Throat Temperature	Ad hoc when patient feels unwell at home

The five different sites are denoted with the letters *A, B, C, D* and *E*. The pilot projects launched for each site are donated with numbers

### Implementation outcomes

#### Adoption

- Out of the 19 sites that had originally signed up to participate, 5 actually launched and used the device, resulting in a site adoption rate of 26%.
- Similarly, out of the 35 projects that were originally proposed, 10 were launched, giving a project adoption rate of 29%.

#### Sustainability

How long the devices were used in each project are summarised in Table 4, alongside whether the projects signed up to the 3-month extension period.

**Table 4:** Time period of device usage for each project that launched

**Project (site)**	**Start month/year**	**End month/year**	**Time used**	**Extension agreed**
**Project 1 (Site A)**	Jul 2021	Jun 2022	11 months	Yes
**Project 2 (Site A)**	Jul 2021	Oct 2021	3 months	No
**Project 3 (Site A)**	Jul 2021	Oct 2021	3 months	No
**Project 4 (Site A)**	Sep 2021	Oct 2021	1 month	No
**Project 5 (Site B)**	Jul 2021	Apr 2022	9 months	Yes
**Project 6 (Site C)**	Jul 2021	Jan 2022	6 months	Yes
**Project 7 (Site C)**	May 2022	Jun 2022	1 month	Yes
**Project 8 (Site D)**	Aug 2021	Oct 2021	2 months	Yes
**Project 9 (Site D)**	Oct 2021	May 2022	7 months	Yes
**Project 10 (Site E)**	Sep 2021	Jun 2022	9 months	Yes

The five different sites are denoted with the letters *A, B, C, D* and *E*. The pilot projects launched for each site are donated with numbers

Only three of the ten projects, each hosted at a separate site, continued to use the device up until the end of the pilot (June 2022). The average time that a project used the device was 5 months, but it should be noted that this is an underestimate as Site C Project 7 (Table 4) started using the device later on in the pilot and was still running at the end of the pilot. Six of the ten projects agreed to take part in the extension period until September 2022. This includes 3 sites that had already launched the project, and three that had expressed an interest but had not been able to launch during the original pilot timelines. This suggests positive perceptions of the device even among sites and projects that had stopped using the device before the original pilot end date.

#### Fidelity

Out of the 598 patient examinations that could have been performed using the device, 376 (62.88%) were recorded as having been done with the device, while 222 (37.12%) were not. Table 5 presents the number of examinations for each type of examination, and whether the device was used.

**Table 5:** Examinations performed by the device for each health check-up

**Variable**	**Total examinations**	**Device used**	**Device not used**
**Lungs**	174	86 (49.42%)	88 (50.57%)
**Temperature**	66	64 (96.97%)	2 (3.03%)
**Heart**	112	56 (50%)	56 (50%)
**Ears**	58	28 (48.28%)	30 (51.72%)
**Throat**	98	75 (76.53%)	23 (23.47%)
**Heart Rate**	38	34 (89.47%)	4 (10.53%)
**Skin**	52	33 (63.46%)	19 (36.54%)

More examinations of patient’s temperature, throat, heart rate, and skin were made using the device than not. The use of the device when examining patient’s heart was the same as alternative methods. For examining patient’s lungs and ears, the device was less likely to be used than other methods available to the health care professional.

#### Feasibility

As can be seen from Table 6, out of *N*=86 clinicians who responded, 14% (*N*=12) reported that they had to reschedule appointments due to issues with the device. This indicates that approximately 1 in 7 appointments were impacted by poor device quality or error.

**Table 6:** Health care professionals’ responses to the ‘repeated due to poor quality?’ question

**Repeated visit due to poor quality (of device)?**	**Yes**	**No**
	12 (14%)	74 (86%)

#### Costs

The estimated total cost of the pilot for the included projects was £920,545. The largest expenses were project management (52%) and device costs (36%). The estimated costs varied from £3270 to £27,389 for projects that were withdrawn from the pilot, and £41,068 to £96,977 for projects that went live. For projects with episode of care data available, the cost per patient ranged from £637 to £9761 and the cost per visit ranged from £479 to £6101. Table 7 provides breakdown of the estimated cost per visit.

**Table 7:** Estimated costs, cost per patient, and cost per visit for the pilot projects

**Project (site)**	**Project planning cost**	**Project management cost**	**Devices cost**	**Patient visits**	**Total project cost**	**Number of patients**	**Number of visits**	**Cost per patient**	**Cost per visit**
**Site A (project 1)**	1006	49,012	3646	464	54,128	85	113	637	479
**Site A (projects 2, 3, 4)**	1006	47,539	18,230	389	67,165	17	28	3951	2399
**Site B (project 5)**	5844	46,480	5469	3423	61,217	39	44	1570	1391
**Site C (project 6)**	6157	32,343	56,842	1634	96,977	16	25	6061	3879
**Site C (project 7)**	12,350	17,565	18,732	160	48,807	5	8	9761	6101
**Site D (project 8)**	3542	40,112	1823	957	46,433	47	67	988	693
**Site D (Project 9)**	4480	33,362	9474	1477	48,793	6	30	8132	1626
**Site E (project 10)**	5555	36,546	18,589	1882	62,572	9	37	6952	1691
**Withdrew**	1006	-	5541	-	6547	-	-	-	-
**Withdrew**	3270	-	-	-	3270	-	-	-	-
**Withdrew**	2461	40,367	26,828	-	69,656	-	-	-	-
**Withdrew**	5738	24,320	11,010	-	41,068	-	-	-	-
**Withdrew**	6264	-	0	-	6264	-	-	-	-
**Withdrew**	8590	15,278	67,079	-	90,946	-	-	-	-
**Withdrew**	5366	-	13,852	-	19,218	-	-	-	-
**Withdrew**	5366	-	-	-	5366	-	-	-	-
**Withdrew**	6972	26,740	36,461	-	70,173	-	-	-	-
**Withdrew**	5838	26,740	11,368	-	43,947	-	-	-	-
**Withdrew**	2012	44,952	3646	-	50,610	-	-	-	-
**Withdrew**	4421	-	22,967	-	27,389	-	-	-	-
**Total**	97,247	481,356	331,558	10,385	920,545	224	352	4110	2615

The five different sites are denoted with the letters *A, B, C, D* and *E*. The pilot projects launched for each site are donated with numbers*.* Where – is used to represent projects that were withdrawn, for which costs were not estimated or missing data. All costs are in 2022 and in sterling pounds

A total of 132 patient appointments were avoided, resulting in potential cost savings to the NHS of £34,828 (Table 8).

**Table 8:** The number of visits potentially avoided and estimated cost savings

**Visit type**	**Number of visits avoided**	**Unit cost of visit**	**Total costs saved**
**GP**	64	£184.00	£11,776.00
**A&E**	2	£185.00	£370.00
**Acute admission**	11	£802.00	£8822.00
**Ambulance**	55	£252.00	£13,860.00
**Total**	132		£34,828.00

## Discussion

Implementation evaluators need to consider the current healthcare context, where staff vacancies are high and staff morale may be low and consider pragmatic methods of data collection first. In this paper, we present an example of how device-downloadable and administrative data was used to evaluate a remote monitoring device pilot study, framed around Proctor et al.’s [21] implementation outcomes framework. A small sample of qualitative interviews was also conducted at some of the participating sites and care settings to provide additional insights but this was not the focus of this paper.

Despite the budget and time constraints, our evaluation was able to measure five out of the eight implementation outcomes using quantitative indicators from device downloads: adoption, sustainability, fidelity, feasibility and cost. By comparison, the scoping review by Willmeroth et al. [29] found no papers using quantitative indicators of sustainability and appropriateness, and cost, feasibility, acceptability and adoption were also infrequently measured.

Even using quantitative indicators, evaluating all eight outcomes may not typically be feasible, as it may require additional administrative records to be kept or other data sources to be identified and linked, creating additional burden. Therefore, it is important to prioritise and focus on the most critical outcomes that align with the goals and objectives of the implementation project. In future research, we recommend sufficient time be allocated at the beginning of a project to engage key stakeholders in identifying the most critical outcomes to be measured, according to the chosen framework. This will allow for prioritisation of outcome measurement; recommended by Proctor et al. [21] and mirroring recommendations for patient and public involvement and engagement more broadly.

The indicators used in this evaluation give insight into implementation challenges. Notably, there were challenges for sites in launching the device. Despite this, there was still a desire to continue collecting data during the extension period for three of the five sites that launched. Additionally, three other sites, which had encountered challenges and were unable to launch during the original pilot timelines, expressed interest in re-joining and launching during the extension period.

Once launched, the feasibility of the device was demonstrated by the low number of care episodes needing to be repeated due to poor device quality (sound/visual/internet). The cost analysis indicated significant up-front cost for the device and staffing, but also potential savings to offset this cost from avoidable in-person appointments. However, the fidelity of the device was called into question by the proportion of examinations performed using the device versus other instruments, indicating that some of the device’s examination functions were not being used. Each of these implementation challenges was further explored through qualitative interviews, not covered in this paper, to give richer insight from which to develop recommendations for scale-up across other sites.

The pragmatics of evaluation design, of which administrative and downloadable data is one such example, is gaining traction in the literature. For instance, Hull et al. [12] reviewed the robustness of survey-based outcome measures which were mapped onto the implementation outcomes framework and scored these measures in terms of their practicality. This was conceptualised as the brevity of the measure, emphasising the importance of measures that are both psychometrically sound (having good reliability, validity and sensitivity to detect change) and brief, to minimise the burden on respondents and improve completion rates. Similarly, the rapid framework approach to the analysis of qualitative data, as outlined by Gale et al. [8], emphasises speed of analysis without sacrificing quality. This approach allows for a more efficient use of researcher time while still producing reliable and valid results. As demonstrated in their study, the rapid analysis yielded highly similar findings to those obtained through their comparative in-depth analysis. This is important as with low robustness, measurement becomes meaningless.

In the evaluation reported here, the reliability and validity of the indicators was considered through discussions with the device manufacturer and the pilot team to check that the variables measured what they appeared to measure, and that they did this reliably. The decision was taken to exclude the penetration outcome measures for both patients and clinicians due to project sites failing to update those records over time, making the data unreliable. This decision would not have been made without the participation of these key stakeholders. The need for valid and reliable measures is as relevant for indicators as it is for surveys and is an area still requiring effort in future research for both types of measures. Willmeroth et al. [29], for example, found only 2 of the 10 studies in their scoping review had indictors which met quality criteria laid down by the National Quality Forum (2017), while Lewis et al. [13] emphasise the need to assess the psychometric robustness of future survey measures. Weiner et al.’s [28] validated measures of feasibility, appropriateness and acceptability provide a good example of how the latter can be done. Nonetheless, for quick turn-around pilot evaluations such as this example, which lasted a year from start to finish, we suggest that the stakeholder engagement process is on par with checking the face and content validity of surveys. Additional robustness checks—for example, identifying other similar measures and then collecting and comparing scores to test convergent and divergent validity, could be particularly time consuming and need to involve additional operational health care staff. This is justifiable and right for large-scale research studies, but likely to be less amendable to smaller-scale pilots, where indicators may vary from project to project, especially those led by health care organisations.

Nonetheless, a limitation of this evaluation is the inability to confidently interpret the indicator data as, for example, high, middle or low adoption/feasibility/fidelity. Future research efforts, thus, should explore how best to interpret indicator data, in a more meaningful way, beyond simply reporting rates. For example, while we have been able to report adoption rates, the confident interpretation and classification of them as ‘high’, ‘moderate’ or ‘low’ has not been possible and is a limitation of this evaluation. When examining other implementation evaluation papers that have been guided by Proctor et al.’s framework (e.g. [9]) to help guide our interpretation, no firm rules were identified. Garner et al. [10] did suggest a cut-off point for interpreting fidelity rates based on evidence linking a particular number of intervention exposures and patient health outcomes, but this was relevant only in the context of their particular evaluation. Stiles et al. [26] examined different methods of calculating penetration rates, offering recommendations for best practice. However, interpretation was not the focus of their paper. Cut-off points or classifications may be less important when monitoring implementation over time or benchmarking, as the results can be interpreted relative to previous measures or other sites. However, clear and meaningful interpretation is essential for effective decision-making and action-planning irrespective of research design.

A further limitation is the lack of comparison across sites, taking into account their context and methods of device usage, which would have provided greater richness. The remit of the evaluation was broad and the timelines tight to include all sites and projects where the device was implemented and to focus on aggregated data, rather than focussing only on, for example, care home settings. The qualitative findings, not covered in this paper, provided more insight into care setting-specific issues and as such, we recommend that future research consider contextual variables a priori that data can be split by, and consider complementing the indicators with some qualitative data.

This paper does not attempt to claim that administrative and routine or downloadable data can solve all challenges of implementation evaluation. Indeed, there are limitations as outlined above. Further, the task of downloading and cleaning data, while reducing burden on frontline health care professionals, may still require involvement of operational staff, such as computing and quality improvement or audit teams. Further insights afforded by in-depth qualitative research and psychometrically robust survey measures such as those developed by Weiner et al. [28] are also incredibly valuable. Nonetheless, we argue that it is important for researchers to first consider using these data sources to measure implementation outcomes rather than relying solely on interviews and surveys.

## Conclusions

This research has provided an example of a rapid evaluation of implementation of a remote monitoring pilot study, framed around Proctor et al.’s [21] implementation outcomes framework and using downloaded and administrative data to measure these outcomes. The results have shown that despite some limitations and challenges, the use of this data can provide valuable insights into the implementation process and success without adding substantially to healthcare staff burden. However, it is important to note that this approach should not entirely replace other methods of data collection, such as qualitative research and psychometrically robust measures where these are feasible. Future research should focus on finding a balance between the use of difference data sources and methods to ensure that implementation evaluations are both efficient and effective. Emphasis should be placed on robust measurement and interpretation of implementation indicators. Overall, this research emphasises the importance of considering and using different data sources in implementation evaluation to gain a comprehensive understanding of the implementation process.

### Supplementary Information

**Additional file 1:** Additional costing methods. **Table S1.** Summary of assumptions used for training and meetings held during the device implementation used in the cost analysis. **Table S2.** Unit costs per type of resource. **Table S3.** Details of projects used in the cost analysis. **Table S4.** Visit types, number of patients and visits, and duration of visits for each project.

**Additional file 2.** Standards for Reporting Implementation Studies: the StaRI checklist for completion.

**Additional file 3.** Audit worksheet organized by manuscript or grant proposal section.

## Data Availability

The dataset is not available due to data sharing agreements.

## Abbreviations

COM-B: Capability, Opportunity, Motivation- Behaviour Model
Yorkshire and Humber AHSN: Yorkshire and Humber Academic Health Science Network
