# Supplementary material for: Evaluating change in a pressured healthcare system: a cross-sectional study of implementation outcomes using routine data indicators and proxies
Source: Implement Sci Commun. 2023 Aug 16;4:96. doi: 10.1186/s43058-023-00471-x (PMC10428631; doi:10.1186/s43058-023-00471-x)
Supplement: Supplementary file 1 — Additional file 1: Additional costing methods. Table S1. Summary of assumptions used for training and meetings held during the device implementation used in the cost analysis. Table S2. Unit costs per type of resource. Table S3. Details of projects used in the cost analysis. Table S4. Visit types, number of patients and visits, and duration of visits for each project. [file 43058_2023_471_MOESM1_ESM.docx]

### **Additional file 1**

### **Additional costing methods**

Costs of the pilot within the Yorkshire and Humber region were estimated from the perspective of the NHS and personal social services. Resources used in the pilot were identified by mapping the processes/steps undertaken to plan and implement the pilot. This was done through project document review, speaking to project staff and a review of care pathways.

#### **Identification of resources**

Resource use was identified and estimated for the pre-implementation and implementation phases of the project. Resources identified in the pre-implementation phase included device procurement (Home device only) and setup, staff times for attending conception/scoping or sensitisation meetings (product demos), training (device training, super-user training, administration support training, and clinician training) and planning meetings. Implementation resources include device subscriptions, staff time for project coordination/management, staff time for attending project meetings, device running costs, staff time for direct patient contact, staff travel time and staff travel cost.

#### **Measurement of resource use**

Costs associated with training and meetings were based on valuing staff time spent attending meetings and training. Although the participants could have attended these in normal working time, we used this approach in order to estimate the opportunity cost of their participation in this intervention. In practice, there were some variations in the number of training sessions or meetings, for example, more product demonstrations were given to individual organisations on request. However, we assumed the same number of training sessions and meetings for all projects due to the unavailability of project-specific data. The exact times for each meeting/training were not available and we assumed it took an average of 1 hour for each meeting/training session (based on information provided by the Yorkshire and Humber AHSN staff). The number of staff taking part in each meeting/training session was only available for the demonstration sessions and we make simplifying assumptions for the rest based on verbal descriptions of project implementation provided by YHAHSN staff. Details on the meetings and training sessions conducted and included in the analysis are provided in Table S1.

**Table S1. Summary of assumptions used for training and meetings held during the device implementation used in the cost analysis.**

| **Activity** | **Details** | **Session duration (hours)** | **Number of participants** |
| --- | --- | --- | --- |
| Project scoping | Not included |  |  |
| Product demonstrations | 1 session per region (3 in total) | 1 | Mean: 25 (range; 20-34) |
| Device training | All project team members (Project manager, ANP/ACP nurse, consultant) | 1 | 3 |
| Super-user training | Project manager (clinical/non-clinical) | 1 | 1 |
| Admin support training | Project manager (clinical/non-clinical) | 1 | 1 |
| Clinician training | All clinical team members (ANP/ACP nurse, consultant) | 1 | 2 |
| Pre-implementation meetings | Weekly meetings from project scoping to go live (Project manager & clinical lead) | 1 | 2 |
| Implementation meetings | Weekly meetings from the project go live (Project manager & clinical lead) | 1 | 2 |

The exact composition of each provider project team was not available (likely to be variable). In consultation with YHAHSN, we assumed the teams to comprise a project manager (clinical or non-clinical), an advanced nurse practitioner (ANP)/a clinical nurse specialist (CNS), a consultant and a registrar for Acute Trusts and a GP, a GP practice nurse, and healthcare assistant for GP practices and care homes. Project coordination/management costs comprise the time spent by the project team members working on the project, estimated to range from 2-7.5 hours per week for the project manager/GP practice nurse, 2-4 hours per week for a consultant/GP, and 2 hours per week for a registrar/healthcare assistant. We also estimated the project manager & clinical lead’s time spent attending weekly meetings (assumed to be 1 hour as previously described) during the entire implementation. In practice, consultants and others would attend these weekly meetings when necessary, however, these were not included due to the unavailability of specific data.

Information on the numbers of each device procured for the pilot was provided by the YHAHSN team.

Direct healthcare professional contacts (consultations) with patients were estimated based on the staff type and the duration of the contact. Healthcare professional contacts were counted directly from visit information obtained from pseudonymised device platform activity data for projects. This information included the device type (home versus pro), type of consultation (direct online consultation, offline examination followed by online consultation/review, and offline examination followed by offline consultation/review), clinician expertise/role (e.g. CNS, GP, Consultant etc), and duration of consultation in minutes. Some data on clinician expertise/roles were missing and some assumptions were made depending on the nature of the project (care homes versus patients supported at home). For GP practices and care homes, we assumed the consultation was done by a healthcare assistant while a Band 7 nurse was assumed for Acute Trusts. All recorded demonstration visits and cancelled appointments were excluded as they are not actual visits following data cleaning rules advised by the YHAHSN.

We assumed offline consultations recorded on the devices were performed by a patient and only required healthcare staff time for reviewing them or performing a follow-on consultation. We assumed offline consultations recorded on the Pro devices were performed by a healthcare professional in face-to-face contact with the patient. We further assumed it was the healthcare professionals who travelled to the consultation and included the relevant travel cost (time and transportation). Visit durations for all offline consultations were not available and were assumed to be 30 minutes.

#### Cost analysis

Staff time was valued by assigning staff groups and unit costs per hour of staff time reported by the Personal Social Services Research Unit (PSSRU). Device costs were estimated using procurement prices negotiated and provided by the project team. Device prices were revised following further negotiations and these prices were used in the sensitivity analysis. To estimate the travel cost for healthcare professionals, we assumed the average travel time to and from the patient’s location was 1 hour and applied a general speed limit of 30 miles per hour to estimate the number of miles travelled in 1 hour (30 miles). We then applied the NHS reimbursement rate for the first 3,500 miles travelled of 56p per mile to estimate the travelling cost. This cost was applied uniformly for all visits assumed to require healthcare staff travel. Unit costs for the different resources used are shown in Table S2.

**Table S2. Unit costs per type of resource**

| **Sector** | **Resource unit** | **Unit Cost (£)** | **Source** |
| --- | --- | --- | --- |
| Acute trust | Hospital-based nurse band 7 | 62 | PSSRU 2021[1] |
| Acute trust | Consultant medical | 123 | PSSRU 2021[1] |
| Acute trust | Specialty registrar | 52 | PSSRU 2021[1] |
| GP practice/Care home | General Practitioner (Cost per hour of patient contact, including direct care) | 217 | PSSRU 2021[1] |
| GP practice/Care home | GP practice nurse | 42 | PSSRU 2021[1] |
| GP practice/Care home | Healthcare assistant | 15 | Assumption |
| All | Staff travel per mile | 0.56 | PSSRU 2020[1] |

We computed the total costs for each project and overall costs for all projects in the pilot using project details provided by YHAHSN. We also calculated the cost per patient and cost per visit for projects with visit data as well as for the overall pilot. All costs are reported in British pounds sterling for 2022.

#### **Cost savings**

Data from the device clinician feedback survey provided by YHAHSN was analysed to determine the number of times a clinician considered that using the device had avoided further healthcare contact for a patient (including a GP visit, A & E visit, acute admission and ambulance use). We applied unit costs to each avoided contact to estimate the potential cost-savings realised from the avoided contacts.

### **Additional tables**

**Table S3. Details of projects used in the cost analysis**

| **Project site (project)** | **ICS Region** | **Organisation type** | **Status** | **Time to go live/withdraw (weeks)** | **Total project duration (weeks)** | **Number of Pro devices** | **Number of Home devices** |
| --- | --- | --- | --- | --- | --- | --- | --- |
| Site A (Project i) | WYH | GP Practice/PCN | Went live | 7.9 | 52.3 | 2 | 0 |
| Site A (Project ii, iii, iv) | WYH | Care Home | Went live | 21.3 | 50.7 | 10 | 0 |
| Not launched/withdrawn/no activity | WYH | Care Home | Withdrawn | 52.4 | - | 2 | 2 |
| Not launched/withdrawn/no activity | HCV | Acute Trust | Withdrawn | 20.6 | - | 0 | 0 |
| Not launched/withdrawn/no activity | WYH | Acute Trust | Went live | 13.7 | 45.3 | 9 | 11 |
| Site D (Project i) | WYH | Acute Trust | Went live | 22.4 | 45.0 | 1 | 0 |
| Site D (Project ii) | WYH | Acute Trust | Went live | 30.0 | 37.4 | 0 | 10 |
| Site D (Project i) | WYH | Acute Trust | Went live | 40.1 | 27.3 | 5 | 2 |
| Not launched/withdrawn/no activity | HCV | Acute Trust | Withdrawn | 44.7 | - | 0 | 0 |
| Site E (Project i) | HCV | Acute Trust | Went live | 39.0 | 41.0 | 5 | 10 |
| Not launched/withdrawn/no activity | WYH | Acute Trust | Went live | 63.1 | 17.1 | 29 | 15 |
| Site B (Project i) | WYH | Acute Trust | Went live | 41.0 | 52.1 | 3 | 0 |
| Not launched/withdrawn/no activity | WYH | Acute Trust | Withdrawn | 37.1 | - | 5 | 5 |
| Not launched/withdrawn/no activity | WYH | Acute Trust | Withdrawn | 37.1 | - | 12 |  |
| Not launched/withdrawn/no activity | HCV | Acute Trust | Went live | 50.4 | 30.0 | 20 | 0 |
| Not launched/withdrawn/no activity | HCV | Acute Trust | Went live | 41.3 | 30.0 | 0 | 12 |
| Site C (Project i) | SYB | Acute Trust | Went live | 10.4 | 50.4 | 2 | 0 |
| Site C (Project i) | SYB | Acute Trust | Went live | 43.9 | 36.3 | 0 | 60 |
| Site C (Project ii , Pro) | SYB | Acute Trust | Went live | 44.0 | 9.9 | 3 | 0 |
| Site C (Project ii, (Home) | SYB | Acute Trust | Went live | 44.0 | 9.9 | 0 | 14 |
| Not launched/withdrawn/no activity | SYB | Acute Trust | Withdrawn | 29.9 | - | 10 | 5 |

The five different sites are denoted with the letters *A, B, C, D* and *E*. The pilot projects launched for each site are donated with roman numerals *i, ii, iii, iv.*

**Table S4. Visit types, number of patients and visits, and duration of visits for each project.**

| **Site** | **Project** | **Visit type** | **Number of patients** | **Number of visits** | **Mean visit duration (minutes)** |
| --- | --- | --- | --- | --- | --- |
| Site A | Project i | Direct online | 84 | 111 | 13.3 |
| Site A | Project i | Offline by HCP to offline | 2 | 2 | 30.0 |
| Site A | Project iii | Direct online | 2 | 3 | 19.7 |
| Site A | Project iii | Offline by HCP to offline | 3 | 4 | 30.0 |
| Site A | Project iv | Direct online | 8 | 10 | 10.3 |
| Site A | Project ii | Direct online | 5 | 8 | 10.4 |
| Site A | Project ii | Offline by HCP to offline | 3 | 3 | 30.0 |
| Site D | Project i | Direct online | 44 | 63 | 7.7 |
| Site D | Project i | Offline by HCP to offline | 2 | 3 | 30.0 |
| Site D | Project i | Offline by patient to offline | 1 | 1 | - |
| Site D | Project ii | Direct online | 3 | 8 | 14.0 |
| Site D | Project ii | Offline by patient to offline | 6 | 19 | - |
| Site D | Project ii | Offline by HCP to online | 1 | 1 | 21.0 |
| Site E | Project i | Direct online | 3 | 10 | 16.1 |
| Site E | Project i | Offline by HCP to offline | 4 | 8 | 30.0 |
| Site E | Project i | Offline by patient to offline | 5 | 19 | - |
| Site B | Project i | Direct online | 1 | 1 | 4.0 |
| Site B | Project i | Offline by HCP to offline | 17 | 19 | 30.0 |
| Site B | Project I | Offline by patient to offline | 22 | 24 | - |
| Site C | Project i | Direct online | 8 | 11 | 11.0 |
| Site C | Project I | Offline by HCP to offline | 9 | 10 | 30.0 |
| Site C | Project i | Offline by patient to offline | 2 | 4 | - |
| Site C | Project ii | Direct online | 3 | 4 | 8.8 |
| Site C | Project ii | Offline by patient to offline | 3 | 4 | - |

The five different sites are denoted with the letters *A, B, C, D* and *E*. The pilot projects launched for each site are donated with roman numerals *i, ii, iii, iv.* Data on the duration of Offline by patient to offline visits was not available but was not considered useful for the analysis as patient/carer costs were excluded

### References

1. Jones, K.C. and A. Burns, Unit costs of health and social care 2021*.* 2021.
