# Supplementary material for: Evaluating change in a pressured healthcare system: a cross-sectional study of implementation outcomes using routine data indicators and proxies
Source: Implement Sci Commun. 2023 Aug 16;4:96. doi: 10.1186/s43058-023-00471-x (PMC10428631; doi:10.1186/s43058-023-00471-x)
Supplement: Supplementary file 3 — Additional file 3. Audit worksheet organized by manuscript or grant proposal section. [file 43058_2023_471_MOESM3_ESM.docx]

Additional file 1.

*Audit worksheet organized by manuscript or grant proposal section.*

| **Circle each implementation outcome that you are investigating. For each circled, complete all items in that column.** | | | | | | | | | | | |
| --- | --- | --- | --- | --- | --- | --- | --- | --- | --- | --- | --- |
|  | | Acceptability | Adoption *✓* | Appropriateness | Cost *✓* | Feasibility *✓* | Fidelity *✓* | Penetration *✓* | Sustainability *✓* | Other outcome not in 2011 taxonomy:  N/A | Other outcome not in 2011 taxonomy:  N/A |
| Uses outcome from 2011 taxonomy but calls it something different. List term in this row. | | N/A | No | N/A | No | No | No | No | No |  |  |
| **Introduction Section** | | | | | | | | | | | |
| 1 | Is each outcome clearly stated in the study aim(s), research question(s) and/or hypothesis(es)? | □ yes  □ no | □ yes  □ no | □ yes  □ no | □ yes  □ no | □ yes  □ no | □ yes  □ no | □ yes  □ no | □ yes  □ no | □ yes  □ no  N/A | □ yes  □ no N/A |
| 2 | Does each implementation outcome have an operational definition in the Introduction? | □ yes *✓*  □ no | □ yes *✓*  □ no | □ yes *✓*  □ no | □ yes *✓*  □ no | □ yes *✓*  □ no | □ yes *✓*  □ no | □ yes *✓*  □ no | □ yes *✓*  □ no | □ yes  □ no N/A | □ yes  □ no N/A |
| 3 | Does the manuscript or grant proposal cite the Proctor et al. 2011 paper, but use a taxonomy term from the paper in a new way? | □ yes  □ explanation for new term usage is provided  □ no *✓* | □ yes  □ explanation for new term usage is provided  □ no *✓* | □ yes  □ explanation for new term usage is provided  □ no *✓* | □ yes  □ explanation for new term usage is provided  □ no *✓* | □ yes  □ explanation for new term usage is provided  □ no *✓* | □ yes  □ explanation for new term usage is provided  □ no *✓* | □ yes  □ explanation for new term usage is provided  □ no *✓* | □ yes  □ explanation for new term usage is provided  □ no *✓* | □ yes  □ explanation for new term usage is provided  □ no N/A | □ yes  □ explanation for new term usage is provided  □ no N/A |
| 4 | Does the Introduction describe how each implementation outcome will be analyzed relative to other constructs in the study aim(s), research question(s) and/or hypothesis(es)?  If yes, check all items that are relevant to your work. | □ yes  □ an independent variable  □ a dependent variable  □ a mediating variable  □ a moderating variable  □ a descriptive variable  □ no  N/A | □ yes *✓*  □ an independent variable  □ a dependent variable  □ a mediating variable  □ a moderating variable  □ a descriptive variable *✓*  □ no | □ yes  □ an independent variable  □ a dependent variable  □ a mediating variable  □ a moderating variable  □ a descriptive variable  □ no  N/A | □ yes *✓*  □ an independent variable  □ a dependent variable  □ a mediating variable  □ a moderating variable  □ a descriptive variable *✓*  □ no | □ yes *✓*  □ an independent variable  □ a dependent variable  □ a mediating variable  □ a moderating variable  □ a descriptive variable *✓*  □ no | □ yes *✓*  □ an independent variable  □ a dependent variable  □ a mediating variable  □ a moderating variable  □ a descriptive variable *✓*  □ no | □ yes *✓*  □ an independent variable  □ a dependent variable  □ a mediating variable  □ a moderating variable  □ a descriptive variable *✓*  □ no | □ yes *✓*  □ an independent variable  □ a dependent variable  □ a mediating variable  □ a moderating variable  □ a descriptive variable *✓*  □ no | □ yes  □ an independent variable  □ a dependent variable  □ a mediating variable  □ a moderating variable  □ a descriptive variable  □ no  N/A | □ yes  □ an independent variable  □ a dependent variable  □ a mediating variable  □ a moderating variable  □ a descriptive variable  □ no  N/A |
| 5 | Does the Introduction state the referent for each implementation outcome?  If yes, check all items that are relevant to your work. | □ yes  □ an intervention □ more than one intervention □ a component of a multicomponent intervention □ an implementation strategy □ more than one implementation strategy □ a component of a multicomponent implementation strategy □ other (see options in manuscript text)  □ no  N/A | □ yes *✓*  □ an intervention *✓* □ more than one intervention □ a component of a multicomponent intervention □ an implementation strategy □ more than one implementation strategy □ a component of a multicomponent implementation strategy □ other (see options in manuscript text)  □ no | □ yes  □ an intervention  □ more than one intervention □ a component of a multicomponent intervention □ an implementation strategy □ more than one implementation strategy □ a component of a multicomponent implementation strategy □ other (see options in manuscript text)  □ no  N/A | □ yes *✓*  □ an intervention *✓* □ more than one intervention □ a component of a multicomponent intervention □ an implementation strategy □ more than one implementation strategy □ a component of a multicomponent implementation strategy □ other (see options in manuscript text)  □ no | □ yes *✓*  □ an intervention *✓* □ more than one intervention □ a component of a multicomponent intervention □ an implementation strategy □ more than one implementation strategy □ a component of a multicomponent implementation strategy □ other (see options in manuscript text)  □ no | □ yes *✓*  □ an intervention *✓* □ more than one intervention □ a component of a multicomponent intervention □ an implementation strategy □ more than one implementation strategy □ a component of a multicomponent implementation strategy □ other (see options in manuscript text)  □ no | □ yes *✓*  □ an intervention *✓* □ more than one intervention □ a component of a multicomponent intervention □ an implementation strategy □ more than one implementation strategy □ a component of a multicomponent implementation strategy □ other (see options in manuscript text)  □ no | □ yes *✓*  □ an intervention *✓* □ more than one intervention □ a component of a multicomponent intervention □ an implementation strategy □ more than one implementation strategy □ a component of a multicomponent implementation strategy □ other (see options in manuscript text)  □ no | □ yes  □ an intervention □ more than one intervention □ a component of a multicomponent intervention □ an implementation strategy □ more than one implementation strategy □ a component of a multicomponent implementation strategy □ other (see options in manuscript text)  □ no  N/A | □ yes  □ an intervention □ more than one intervention □ a component of a multicomponent intervention □ an implementation strategy □ more than one implementation strategy □ a component of a multicomponent implementation strategy □ other (see options in manuscript text)  □ no  N/A |
| **Methods Section** | | | | | | | | | | | |
| 1 | Does each implementation outcome have an operational definition in the Methods?  If yes, is it consistent with the definition given in the Introduction? | □ yes  □ is consistent with Introduction item #2  □ inconsistent with Introduction item #2  □ no | □ yes  □ is consistent with Introduction item #2  □ inconsistent with Introduction item #2  □ no | □ yes  □ is consistent with Introduction item #2  □ inconsistent with Introduction item #2  □ no | □ yes  □ is consistent with Introduction item #2  □ inconsistent with Introduction item #2  □ no | □ yes  □ is consistent with Introduction item #2  □ inconsistent with Introduction item #2  □ no | □ yes  □ is consistent with Introduction item #2  □ inconsistent with Introduction item #2  □ no | □ yes  □ is consistent with Introduction item #2  □ inconsistent with Introduction item #2  □ no | □ yes  □ is consistent with Introduction item #2  □ inconsistent with Introduction item #2  □ no | □ yes  □ is consistent with Introduction item #2  □ inconsistent with Introduction item #2  □ no | □ yes  □ is consistent with Introduction item #2  □ inconsistent with Introduction item #2  □ no |
| 2 | Are implementation outcomes combined or merged in the Methods to create a new variable? | □ yes  □ explanation or rationale provided    □ no N/A | □ yes  □ explanation or rationale provided  □ no *✓* | □ yes  □ explanation or rationale provided  □ no  N/A | □ yes  □ explanation or rationale provided  □ no *✓* | □ yes  □ explanation or rationale provided  □ no *✓* | □ yes  □ explanation or rationale provided  □ no *✓* | □ yes  □ explanation or rationale provided  □ no *✓* | □ yes  □ explanation or rationale provided  □ no *✓* | □ yes  □ explanation or rationale provided  □ no  N/A | □ yes  □ explanation or rationale provided  □ no N/A |
| 3 | Do the Methods clearly state how each implementation outcome will be analyzed relative to other constructs in the study aim(s), research question(s) and/or hypothesis(es)?  If yes, is it consistent with what is described in the Introduction? | □ yes  □ is consistent with Introduction item #4  □ inconsistent with Introduction item #4  □ no  N/A | □ yes  □ is consistent with Introduction item #4 *✓*  □ inconsistent with Introduction item #4  □ no | □ yes  □ is consistent with Introduction item #4  □ inconsistent with Introduction item #4  □ no  N/A | □ yes  □ is consistent with Introduction item #4 *✓*  □ inconsistent with Introduction item #4  □ no | □ yes  □ is consistent with Introduction item #4 *✓*  □ inconsistent with Introduction item #4  □ no | □ yes  □ is consistent with Introduction item #4 *✓*  □ inconsistent with Introduction item #4  □ no | □ yes  □ is consistent with Introduction item #4 *✓*  □ inconsistent with Introduction item #4  □ no | □ yes  □ is consistent with Introduction item #4 *✓*  □ inconsistent with Introduction item #4  □ no | □ yes  □ is consistent with Introduction item #4  □ inconsistent with Introduction item #4  □ no  N/A | □ yes  □ is consistent with Introduction item #4  □ inconsistent with Introduction item #4  □ no  N/A |
| 4 | Does the manuscript or grant proposal report psychometric properties or citations for all standardized instruments used to measure each implementation outcome? | □ yes  □ no  N/A | □ yes  □ no  N/A | □ yes  □ no  N/A | □ yes  □ no  N/A | □ yes  □ no  N/A | □ yes  □ no  N/A | □ yes  □ no  N/A | □ yes  □ no  N/A | □ yes  □ no  N/A | □ yes  □ no  N/A |
| 5 | Does each implementation outcome measured have a clear referent?  If yes, is it consistent with the referent given in the Introduction? | □ yes  □ is consistent with Introduction item #5  □ inconsistent with Introduction item #5  □ no N/A | □ yes  □ is consistent with Introduction item #5 *✓*  □ inconsistent with Introduction item #5  □ no | □ yes  □ is consistent with Introduction item #5  □ inconsistent with Introduction item #5  □ no N/A | □ yes  □ is consistent with Introduction item #5 *✓*  □ inconsistent with Introduction item #5  □ no | □ yes  □ is consistent with Introduction item #5 *✓*  □ inconsistent with Introduction item #5  □ no | □ yes  □ is consistent with Introduction item #5 *✓*  □ inconsistent with Introduction item #5  □ no | □ yes  □ is consistent with Introduction item #5 *✓*  □ inconsistent with Introduction item #5  □ no | □ yes  □ is consistent with Introduction item #5 *✓*  □ inconsistent with Introduction item #5  □ no | □ yes  □ is consistent with Introduction item #5  □ inconsistent with Introduction item #5  □ no N/A | □ yes  □ is consistent with Introduction item #5  □ inconsistent with Introduction item #5  □ no N/A |
| 6 | Will the same outcome be measured and assessed across multiple referents/ “things” (e.g., fidelity of an intervention and of a strategy)? | □ yes  □ no  N/A | □ yes *✓*  □ no | □ yes  □ no  N/A | □ yes *✓*  □ no | □ yes  □ no *✓* | □ yes  □ no *✓* | □ yes *✓*  □ no | □ yes *✓*  □ no | □ yes  □ no  N/A | □ yes  □ no  N/A |
| 7 | For each implementation outcome, what type of data will be collected? See options in manuscript text. | □ quantitative data (list types here):  □ qualitative data (list types here):   N/A | □ quantitative data (list types here): *✓*  Administrative records  □ qualitative data (list types here): | □ quantitative data (list types here):  □ qualitative data (list types here):  N/A | □ quantitative data (list types here): *✓*  Device downloadable data  □ qualitative data (list types here): | □ quantitative data (list types here): *✓*   Device downloadable data  □ qualitative data (list types here): | □ quantitative data (list types here): *✓*  Device downloadable data  Administration records  □ qualitative data (list types here): | □ quantitative data (list types here): *✓*  Administrative data  Device downloadable data  □ qualitative data (list types here): | □ quantitative data (list types here): *✓*  Administrative data  □ qualitative data (list types here): | □ quantitative data (list types here):  □ qualitative data (list types here):  N/A | □ quantitative data (list types here):  □ qualitative data (list types here):  N/A |
| 8 | Will a mixed methods design be used to assess the implementation outcome? | □ yes  □ mixed methods design explained *✓*  □ mixed methods design not explained  □ no | □ yes  □ mixed methods design explained *✓*  □ mixed methods design not explained  □ no | □ yes  □ mixed methods design explained *✓*  □ mixed methods design not explained  □ no | □ yes  □ mixed methods design explained *✓*  □ mixed methods design not explained  □ no | □ yes  □ mixed methods design explained *✓*  □ mixed methods design not explained  □ no | □ yes  □ mixed methods design explained *✓*  □ mixed methods design not explained  □ no | □ yes  □ mixed methods design explained *✓*  □ mixed methods design not explained  □ no | □ yes  □ mixed methods design explained *✓*  □ mixed methods design not explained  □ no | □ yes  □ mixed methods design explained  □ mixed methods design not explained  □ no  N/A | □ yes  □ mixed methods design explained  □ mixed methods design not explained  □ no  N/A |
| 9 | Who will provide/report data for each implementation outcome?  Check all items that are relevant to your work. | □ client/patient  □ frontline provider  □ supervisor/middle manager  □ administrator/ executive leader  □ policymaker  □ other (list):  N/A | □ client/patient  □ frontline provider  □ supervisor/middle manager  □ administrator/ executive leader *✓*  □ policymaker  □ other (list): | □ client/patient  □ frontline provider  □ supervisor/middle manager  □ administrator/ executive leader  □ policymaker  □ other (list):  N/A | □ client/patient  □ frontline provider *✓*  □ supervisor/middle manager  □ administrator/ executive leader *✓*  □ policymaker  □ other (list): | □ client/patient  □ frontline provider  □ supervisor/middle manager  □ administrator/ executive leader  □ policymaker  □ other (list): *✓*  Device | □ client/patient  □ frontline provider  □ supervisor/middle manager  □ administrator/ executive leader *✓*  □ policymaker  □ other (list): *✓*  Device | □ client/patient  □ frontline provider  □ supervisor/middle manager  □ administrator/ *✓*executive leader  □ policymaker  □ other (list): *✓*  Device | □ client/patient  □ frontline provider  □ supervisor/middle manager  □ administrator/ *✓*executive leader  □ policymaker  □ other (list): | □ client/patient  □ frontline provider  □ supervisor/middle manager  □ administrator/ executive leader  □ policymaker  □ other (list): N/A | □ client/patient  □ frontline provider  □ supervisor/middle manager  □ administrator/ executive leader  □ policymaker  □ other (list): N/A |
| 10 | Will an individual provide implementation outcome data for a higher level of analysis (e.g., team or organization)? | □ yes  □ the rationale for this data collection decision is clear  □ the rationale is unclear or not stated  □ no  N/A | □ yes  □ the rationale for this data collection *✓*decision is clear  □ the rationale is unclear or not stated  □ no | □ yes  □ the rationale for this data collection decision is clear  □ the rationale is unclear or not stated  □ no  N/A | □ yes  □ the rationale for this data collection *✓*decision is clear  □ the rationale is unclear or not stated  □ no | □ yes  □ the rationale for this data collection decision is clear  □ the rationale is unclear or not stated  □ no *✓* | □ yes  □ the rationale for this data collection *✓*decision is clear  □ the rationale is unclear or not stated  □ no | □ yes  □ the rationale for this data collection *✓*decision is clear  □ the rationale is unclear or not stated  □ no | □ yes  □ the rationale for this data collection *✓*decision is clear  □ the rationale is unclear or not stated  □ no | □ yes  □ the rationale for this data collection decision is clear  □ the rationale is unclear or not stated  □ no N/A | □ yes  □ the rationale for this data collection decision is clear  □ the rationale is unclear or not stated  □ no  N/A |
| 11 | Does the Methods section report the level of aggregation or analysis for each implementation outcome? | □ yes  □ individual level  □ team or group level  □ organizational level  □ system or community level  □ other:  □ no  □ not applicable *✓*  □ should be included | □ yes  □ individual level  □ team or group level  □ organizational level *✓*  □ system or community level  □ other:  □ no  □ not applicable  □ should be included | □ yes  □ individual level  □ team or group level  □ organizational level  □ system or community level  □ other:  □ no  □ not applicable  □ should be included  N/A | □ yes  □ individual level  □ team or group level  □ organizational level *✓*  □ system or community level  □ other:  □ no  □ not applicable  □ should be included | □ yes  □ individual level  □ team or group level  □ organizational level *✓*  □ system or community level  □ other:  □ no  □ not applicable  □ should be included | □ yes  □ individual level  □ team or group level  □ organizational level *✓*  □ system or community level  □ other:  □ no  □ not applicable  □ should be included | □ yes  □ individual level  □ team or group level  □ organizational level *✓*  □ system or community level  □ other:  □ no  □ not applicable  □ should be included | □ yes  □ individual level  □ team or group level  □ organizational level *✓*  □ system or community level  □ other:  □ no  □ not applicable  □ should be included | □ yes  □ individual level  □ team or group level  □ organizational level  □ system or community level  □ other:  □ no  □ not applicable  □ should be included  N/A | □ yes  □ individual level  □ team or group level  □ organizational level  □ system or community level  □ other:  □ no  □ not applicable  □ should be included  N/A |
| 12 | At what phase(s) or stage(s) of implementation will each implementation outcome be measured?  Check all items that are relevant to your work. | □ preparation, exploration, pre-implementation  □ active implementation  □ sustainment, maintenance  □ not stated  N/A | □ preparation, exploration, pre-implementation  □ active implementation *✓*  □ sustainment, maintenance  □ not stated | □ preparation, exploration, pre-implementation  □ active implementation  □ sustainment, maintenance  □ not stated  N/A | □ preparation, exploration, pre-implementation  □ active implementation *✓*  □ sustainment, maintenance  □ not stated | □ preparation, exploration, pre-implementation  □ active implementation *✓*  □ sustainment, maintenance  □ not stated | □ preparation, exploration, pre-implementation  □ active implementation *✓*  □ sustainment, maintenance  □ not stated | □ preparation, exploration, pre-implementation  □ active implementation *✓*  □ sustainment, maintenance  □ not stated | □ preparation, exploration, pre-implementation  □ active implementation *✓*  □ sustainment, maintenance  □ not stated | □ preparation, exploration, pre-implementation  □ active implementation  □ sustainment, maintenance  □ not stated  N/A | □ preparation, exploration, pre-implementation  □ active implementation  □ sustainment, maintenance  □ not stated  N/A |
| 13 | Are the measurement time points specified? | □ yes  □ once, cross-sectional  □ twice, pre-post  □ three or more times  □ no  N/A | □ yes  □ once, cross-sectional *✓*  □ twice, pre-post  □ three or more times  □ no | □ yes  □ once, cross-sectional  □ twice, pre-post  □ three or more times  □ no  N/A | □ yes  □ once, cross-sectional *✓*  □ twice, pre-post  □ three or more times  □ no | □ yes  □ once, cross-sectional *✓*  □ twice, pre-post  □ three or more times  □ no | □ yes  □ once, cross-sectional *✓*  □ twice, pre-post  □ three or more times  □ no | □ yes  □ once, cross-sectional *✓*  □ twice, pre-post  □ three or more times  □ no | □ yes  □ once, cross-sectional *✓*  □ twice, pre-post  □ three or more times  □ no | □ yes  □ once, cross-sectional *✓*  □ twice, pre-post  □ three or more times  □ no | □ yes  □ once, cross-sectional *✓*  □ twice, pre-post  □ three or more times  □ no |
| 14 | Is rationale provided for the number of measurement timepoints? | □ yes  □ no  N/A | □ yes  □ no  N/A | □ yes  □ no  N/A | □ yes  □ no  N/A | □ yes  □ no  N/A | □ yes  □ no  N/A | □ yes  □ no  N/A | □ yes  □ no  N/A | □ yes  □ no  N/A | □ yes  □ no  N/A |
| **Results Section** | | | | | | | | | | | |
| 1 | Are multiple implementation outcomes combined or merged in the Results to create a new variable?  If yes, is it consistent with what was explained in the Methods? | □ yes  □ is consistent with Methods item #2  □ inconsistent with Methods item #2  □ no  N/A | □ yes  □ is consistent with Methods item #2  □ not explained in Methods  □ no *✓* | □ yes  □ is consistent with Methods item #2  □ not explained in Methods  □ no  N/A | □ yes  □ is consistent with Methods item #2  □ not explained in Methods  □ no *✓* | □ yes  □ is consistent with Methods item #2  □ not explained in Methods  □ no *✓* | □ yes  □ is consistent with Methods item #2  □ not explained in Methods  □ no *✓* | □ yes  □ is consistent with Methods item #2  □ not explained in Methods  □ no *✓* | □ yes  □ is consistent with Methods item #2  □ not explained in Methods  □ no *✓* | □ yes  □ is consistent with Methods item #2  □ not explained in Methods  □ no  N/A | □ yes  □ is consistent with Methods item #2  □ not explained in Methods  □ no  N/A |
| 2 | For each finding presented in the Results, is the referent for each implementation outcome clearly stated?  If yes, is it consistent with what was stated in the Introduction and Methods? | □ yes  □ is consistent with Introduction item #5  □ is consistent with Methods item #5  □ inconsistent with previous section(s)  □ no  N/A | □ yes  □ is consistent with Introduction item #5  □ is consistent with Methods item #5*✓*  □ inconsistent with previous section(s)  □ no | □ yes  □ is consistent with Introduction item #5  □ is consistent with Methods item #5  □ inconsistent with previous section(s)  □ no  N/A | □ yes  □ is consistent with Introduction item #5  □ is consistent with Methods item #5*✓*  □ inconsistent with previous section(s)  □ no | □ yes  □ is consistent with Introduction item #5  □ is consistent with Methods item #5*✓*  □ inconsistent with previous section(s)  □ no | □ yes  □ is consistent with Introduction item #5  □ is consistent with Methods item #5 *✓*  □ inconsistent with previous section(s)  □ no | □ yes  □ is consistent with Introduction item #5  □ is consistent with Methods item #5 *✓*  □ inconsistent with previous section(s)  □ no | □ yes  □ is consistent with Introduction item #5  □ is consistent with Methods item #5*✓*  □ inconsistent with previous section(s)  □ no | □ yes  □ is consistent with Introduction item #5  □ is consistent with Methods item #5  □ inconsistent with previous section(s)  □ no | □ yes  □ is consistent with Introduction item #5  □ is consistent with Methods item #5  □ inconsistent with previous section(s)  □ no |
| 3 | If the same implementation outcome is assessed across different referents, are findings reported for each? | □ yes  □ no  □ findings missing for this referent (list here):  N/A | □ yes*✓*  □ no  □ findings missing for this referent (list here): | □ yes  □ no  □ findings missing for this referent (list here):  N/A | □ yes*✓*  □ no  □ findings missing for this referent (list here): | □ yes  □ no  □ findings missing for this referent (list here):  N/A | □ yes  □ no  □ findings missing for this referent (list here):  N/A | □ yes  □ no*✓*  □ findings missing for this referent (list here):  Gave reason to why results were not reported for one. | □ yes*✓*  □ no  □ findings missing for this referent (list here): | □ yes  □ no  □ findings missing for this referent (list here): | □ yes  □ no  □ findings missing for this referent (list here): |
| **Discussion Section** | | | | | | | | | | | |
| 1 | Are multiple implementation outcomes combined or merged in the Discussion to create a new variable?  If yes, is it consistent with what was stated in the Methods and Results? | □ yes  □ is consistent with Methods item #2  □ is consistent with Results item #1  □ inconsistent with previous section(s)  □ no  N/A | □ yes  □ is consistent with Methods item #2  □ is consistent with Results item #1  □ inconsistent with previous section(s)  □ no*✓* | □ yes  □ is consistent with Methods item #2  □ is consistent with Results item #1  □ inconsistent with previous section(s)  □ no  N/A | □ yes  □ is consistent with Methods item #2  □ is consistent with Results item #1  □ inconsistent with previous section(s)  □ no*✓* | □ yes  □ is consistent with Methods item #2  □ is consistent with Results item #1  □ inconsistent with previous section(s)  □ no*✓* | □ yes  □ is consistent with Methods item #2  □ is consistent with Results item #1  □ inconsistent with previous section(s)  □ no*✓* | □ yes  □ is consistent with Methods item #2  □ is consistent with Results item #1  □ inconsistent with previous section(s)  □ no*✓* | □ yes  □ is consistent with Methods item #2  □ is consistent with Results item #1  □ inconsistent with previous section(s)  □ no*✓* | □ yes  □ is consistent with Methods item #2  □ is consistent with Results item #1  □ inconsistent with previous section(s)  □ no  N/A | □ yes  □ is consistent with Methods item #2  □ is consistent with Results item #1  □ inconsistent with previous section(s)  □ no  N/A |
| 2 | For each finding discussed, is the referent for each implementation outcome clearly stated?  If yes, is it consistent with what was stated in the Introduction, Methods, and Results? | □ yes  □ is consistent with Introduction item #5  □ is consistent with Methods item #5  □ is consistent with Results items #2 and #3  □ inconsistent with previous section(s)  □ no  N/A | □ yes  □ is consistent with Introduction item #5  □ is consistent with Methods item #5  □ is consistent with Results items #2 and #3  □ inconsistent with previous section(s)  □ no | □ yes  □ is consistent with Introduction item #5  □ is consistent with Methods item #5  □ is consistent with Results items #2 and #3  □ inconsistent with previous section(s)  □ no N/A | □ yes  □ is consistent with Introduction item #5  □ is consistent with Methods item #5  □ is consistent with Results items #2 and #3  □ inconsistent with previous section(s)  □ no | □ yes  □ is consistent with Introduction item #5  □ is consistent with Methods item #5  □ is consistent with Results items #2 and #3  □ inconsistent with previous section(s)  □ no | □ yes  □ is consistent with Introduction item #5  □ is consistent with Methods item #5  □ is consistent with Results items #2 and #3  □ inconsistent with previous section(s)  □ no | □ yes  □ is consistent with Introduction item #5  □ is consistent with Methods item #5  □ is consistent with Results items #2 and #3  □ inconsistent with previous section(s)  □ no | □ yes  □ is consistent with Introduction item #5  □ is consistent with Methods item #5  □ is consistent with Results items #2 and #3  □ inconsistent with previous section(s)  □ no | □ yes  □ is consistent with Introduction item #5  □ is consistent with Methods item #5  □ is consistent with Results items #2 and #3  □ inconsistent with previous section(s)  □ no | □ yes  □ is consistent with Introduction item #5  □ is consistent with Methods item #5  □ is consistent with Results items #2 and #3  □ inconsistent with previous section(s)  □ no |
